# Molar–Incisor Hypomineralization Is Associated with the Prevalence of Thinness among Schoolchildren in Communities with Different Fluoride Levels in the Drinking Water

**DOI:** 10.1155/2024/6212877

**Published:** 2024-07-12

**Authors:** Alvaro García Pérez, Teresa Villanueva Gutiérrez, Alvaro Edgar González-Aragón Pineda, Karla Lizbeth Murillo Santos, Nora Guillermina Pérez Pérez

**Affiliations:** ^1^ Laboratory of Public Health Research Faculty of Higher Studies (FES) National Autonomous University of Mexico (UNAM), Iztacala, Mexico; ^2^ Health Care Department Metropolitan Autonomous University-Xochimilco, Mexico City, Mexico; ^3^ Pediatric Stomatology Specialties Faculty of Higher Studies (FES) Iztacala National Autonomous University of Mexico (UNAM), Mexico City, Mexico

## Abstract

**Objective:**

To examine the association between molar–incisor hypomineralization (MIH) and the prevalence of thinness among Mexican schoolchildren in communities with different fluoride levels in the drinking water.

**Methods:**

A cross-sectional study on Mexican children (*n* = 488) selected from two communities presenting different concentrations of fluoride in the drinking water (1.0–1.40 ppm/F). The World Health Organization (WHO) growth standards were used to calculate BMI-for-age *z*-scores, with BMI *z*-score cutoff points of <−2.0, >+1.0, >+2.0 recommended for defining thinness, being overweight, and obesity. The presence and severity of MIH were evaluated using the European Academy of Paediatric Dentistry (EAPD) criteria. Multiple logistic regression analyses were used to assess the association, adjusting for confounders.

**Results:**

The proportion of children presenting thinness, being overweight, and obesity was 8.2%, 23.6%, and 28.7%, respectively, while 21.5% of the schoolchildren had MIH, classified, by severity, as 9.6% mild, 6.4% moderate, and 5.5% severe. Of those schoolchildren presenting thinness, 16.2% had MIH, and only 6.0% did not (*p*  < 0.001). Finally, schoolchildren presenting thinness were more likely to present MIH (OR = 2.76 (CI 95% 1.33–5.73); *p*=0.006) than children with a normal BMI.

**Conclusion:**

The present study found a relationship between thinness and the presence of MIH in schoolchildren, indicating the need for strategies and interventions aimed at preventing and controlling micronutrient deficiencies in the child population.

## 1. Introduction

Globally, nutrition continues to be an important factor in maintaining good levels of health in the child population, although it is yet to be adequately integrated into healthcare services and systems, which do not recognize its importance to achieving positive changes in health, growth, childhood development, and survival [1]. Different ages require specific quantities of micronutrients, which are essential for multiple functions that interact during child growth and development. A lack of micronutrients presents with greater frequency in developing countries as a consequence of insufficient food, overeating, or an adverse combination of the two, as well as a low availability of nutrients and other causes [2]. Similarly, micronutrient deficiencies in childhood have been associated with socioeconomic disadvantage, childhood malnutrition, and chronic diseases, thus negatively impacting on quality of life [3, 4].

Childhood malnutrition continues to be a public health problem in developing countries and one of the main causes of childhood morbidity and mortality [5]. The *Encuesta Nacional de Salud y Nutrición* 2021 (ENSANUT, or the National Health and Nutrition Examination Survey) found, in Mexican children under 5 years old, a prevalence of 12.6% low-height individuals, 1.5% emaciated individuals, 3.7% underweight individuals, and 7.8% overweight or obese individuals [6]. Nutritional disorders can affect any system of the body and have been associated with the risk of infection, deficient growth related to delayed mental development, poor academic performance, and reduced intellectual capacity [7]. It has also been observed that the populations presenting malnutrition and chronic diseases also present higher percentages of enamel defects [8].

Developmental enamel defects (DDE) are alterations that occur during the formation of the enamel and can manifest as enamel hypoplasia, molar–incisor hypomineralization (MIH), amelogenesis imperfecta, and dental fluorosis [9]. This relationship between DDE and malnutrition occurs due to the perturbation of ameloblast function during the secretory phase of amelogenesis [10, 11]. MIH is a qualitative enamel defect that mainly affects the first molars and permanent incisors and is characterized by marked opacities that range in color from white to yellow/brown. Severity is distributed asymetrically, while fractures are produced by the force of mastication due to the fragility of the affected enamel [12]. Currently, the etiology of MIH has not been well established, although prenatal or early childhood health factors such as genetic factors, being underweight at birth, dioxins, fever, infections, respiratory problems, the use of antibiotics, and malnutrition, are suspected [13].

Evaluating growth and nutritional status during infancy is of great importance because it enables the early identification of nutritional alterations, adequate diagnosis, and timely treatment. The patterns of childhood growth stipulated by the WHO propose measurements of childhood growth that are standardized by age using *z*-scores [14]. For example, the WHO, using the *z*-score of the body mass index (BMI), which corresponds to age (BMI-for-age *z*-scores), recommends the cutoff points <−2.0, >+1.0, and >2.0 to classify 5–19-year-old children as thin, overweight, and obese, respectively [15]. Thinness can be a marker of malnutrition [16] and can lead to the inhibition of physical and intellectual development [17]. Thinness in children can result from different factors, such as poor dietary habits, deficiencies in essential micronutrients, and the use of adult food supplements that reduce the absorption of vitamins and minerals, among others [18]. On the other hand, thinness in school-age children and its relationship with MIH have been subject to little research. On a global level, a large amount of research has been conducted on the prevalence of childhood obesity, although a lesser amount has been conducted to evaluate thinness. Consequently, evaluating the relationship between these two variables in the child population would help to generate nutritional strategies and public health programs, such as those focusing on nutritional education and healthy dietary behavior, that aim to reduce the prevalence of thinness in school-age children. The present study sought to examine the association between MIH and the prevalence of thinness in Mexican schoolchildren in communities with different fluoride levels in the drinking water. We hypothesized that children who are thin will have a higher probability of presenting MIH.

## 2. Material and Methods

The present study was carried out in adherence with the guidelines set out in the Strengthening the Reporting of Observational Studies in Epidemiology (STROBE) statement. The research protocol was reviewed and approved by the Ethics Committee of the Faculty of Higher Studies Iztacala at the National Autonomous University of Mexico (CE/FESI/062023/1619). Both the authorities of the public primary schools and the parents of the schoolchildren were informed of the protocol, with those parents who agreed to their children's participation signing the informed consent form, while the children authorized their participation by giving their informed consent.

### 2.1. Study Design

The present cross-sectional study was conducted from October 01, 2022, to March 30, 2023. According to the annual report on the situation of poverty and social backwardness 2024 in Mexico, the two study sites are located in the municipality of Ayala in the state of Morelos. The municipality has a total population of 93,823 inhabitants (48.4% men vs. 51.6% women). In terms of the socioeconomic indicators, 26.8% of participants do not have access to basic household services (access to drinking water, sanitary drainage, and electricity), 26.9% do not have access to food, 20.7% present educational lag, 15.1% did not have access to healthcare services, and 53.3% were living in moderate to extreme poverty. Between 2016 and 2018, the Gini coefficient in Morelos [19] went from 0.437 to 0.429, a reduction of 1.9%, which meant a lower level of inequality.

Fluoride concentrations in the drinking water at both sites before starting data collection were determined via the use of a specific electrode (Thermo Scientific Orion Star ™, Waltham, MA, USA), while samples were analyzed in accordance with the Official Mexican Standard (NMX-AA-077-SCFI-2001). Water fluoridation in communities is natural. The fluoride level of the drinking water in the two communities ranged from 1.0 to 1.40 ppm/F.

The *inclusion criteria* for the study were as follows: schoolchildren aged from 7 to 12 years old who attended public elementary schools; either gender; written authorization to participate; the four upper and lower incisors and the first four permanent molars fully erupted; and the parents/guardians of the participant residing at the same address. The *exclusion criteria* were as follows: schoolchildren who had lived at different residences for more than 6 months during their first 7 years of life; the presence of orthodontic attachments that prevented the examination of the tooth surface; or failure to cooperate during the oral examination.

### 2.2. Independent Variable: Nutritional Status

The present study used the WHO *z*-scores to classify the nutritional status of the schoolchildren. The WHO growth standards were selected, as they are meant to reflect optimal growth in children and are recommended for use in the age group of interest in Mexico. The present study classified a child as underweight if the weight-for-age WHO *z*-score was <−2 SD, overweight >+1SD, and obesity >+2SD. The low height-for-age *z*-score was <−2 SD. BMI-for-age *z*-score cutoff points of <−2.0 SD, >+1.0 SD, and >+2.0 SD were used to classify schoolchildren as thinness, overweight, and obesity, respectively [20]. Using this variable provides us with a reference of individuals of that age with respect to population. A standardized nutritionist was hired to conduct the anthropometry to obtain the bodyweight and height of the schoolchildren, with weight measured using a segmental body composition monitor (Tanita BC-568, Tokio, Japan) and standing height was measured using a portable stadiometer (SECA 213, Hamburg, Germany). The WHO AnthroPlus program, which was designed for use with children and adolescents aged from 5 to 19 years of age, was applied to calculate the age- and gender-specific *z*-scores for height and weight [21].

### 2.3. Outcome Variable: MIH

The evaluation of MIH included the inspection of vestibular, occlusal/incisal, and palatal surfaces of all the erupted permanent molars and incisors, with the results classified according to the criteria set out by the European Academy of Paediatric Dentistry (EAPD) [22]. A child was classified as having MIH when any of the first permanent molars showed signs of MIH. The severity of MIH presented by each child was defined by the most severe defect observed in the first permanent molars or permanent incisors and classified as mild, moderate, or severe.

### 2.4. Covariates

The following variables were used as potential confounders and adjusted in the model: age in years (7–8 years, 9–10 years, and 11–12 years); gender (boy/girl); and oral hygiene, as evaluated via the Simplified Oral Hygiene Index (OHI-S). The index comprises debris and calculus scores for selected tooth surfaces. The buccal and lingual surfaces of six permanent index teeth were examined, while oral hygiene was classified as either poor (OHI-S ≥ 2 score) or good (OHI-S < 2 score) [23], and the F water concentration corresponded to 1.00/1.40 ppm/F.

### 2.5. Study Size

Nonprobability sampling was carried out for convenience at two study sites selected in the northern and central sections of the study area, with all those pupils aged between 7 and 12 years invited to participate. The sample size was calculated to detect an odds ratio (OR) = 2.0 with 80% power, an alpha of 0.05, and a probability of 0.30 for MIH. The calculation used data obtained from two previous studies [24, 25]. The sample size, based on the identification of the presence of MIH, was 411 school-age children. Based on the calculation used for the required sample size, a total of 520 informed consent forms for participating in the study were given to the school-age children, with 488 forms then signed by their parents (response rate: 94%).

### 2.6. Clinical Oral Examination

The clinical oral evaluations were conducted by two dentists using dental mirrors, a WHO probe, and artificial light, with the child's teeth brushed prior to the evaluation, in the two public schools selected. The examination of the participating schoolchildren's oral cavity adhered to the corresponding infection control standards. The two examiners participated in a training and calibration exercise, which consisted of two steps (theoretical and clinical) using the MIH index and the OHI-S, while their inter- and intra-examiner agreement for MIH corresponded to a Cohen's kappa coefficient of >82% and an OHI-S > 0.85, respectively. To record information, the OHI-S was first collected, then the children brushed their teeth with supervision, and, finally, MIH evaluation was carried out.

### 2.7. Infection Control Practices for COVID-19

Due to the characteristics of the oral examination conducted in the primary schools, the school-age groups were highly vulnerable to infection with severe acute respiratory syndrome-COVID-19 2 (SARS-CoV−2) [26]. For this reason, standard protection measures were implemented for the oral examination conducted in the schools. These included the maintenance of good hand hygiene and disinfection of all surfaces used using Lysol™, while sodium hypochlorite was used to clean and disinfect the work tables used in each classroom. After the oral examination had been conducted on each child, the work tables were subject to a deep clean, and the dental instruments were then cleaned, sterilized, and stored. The personal protection equipment for the dentists comprised N-95 masks, single-use latex gloves, surgical gowns, and goggles or face shields.

## 3. Statistical Analysis

All analyses were conducted using the Stata 15 software (StataCorp, College Station, TX, USA). Cross-tabulations using Pearson's chi-square test were applied to describe associations between covariates by MIH. The association between nutritional status and MIH was tested via a multiple logistic regression model, in which the dependent variable was MIH and the independent variables were gender, number of erupted teeth, fluoride concentration in the drinking water, oral hygiene, and BMI-for-age *z*-scores. Age was excluded from the model because of its collinearity with the BMI. The OR and 95% confidence interval (95% CI) were calculated, while the model diagnostic tests were conducted using the Hosmer–Lemeshow goodness of fit test. Values of *p*  ≤ 0.05 were considered statistically significant.

## 4. Results

A total of 488 schoolchildren aged 7–12 years, with a mean age of 9.5 (±1.22), were included in the present study, while the percentages of boys and girls examined were 52.3% and 47.7%, respectively, with no statistically significant differences between the mean age found by gender (*p* =0.140). The findings obtained by the present study reveal 56.6% of participants to have had poor oral hygiene. The mean number of erupted permanent teeth was 23.8 (±7.01), while the prevalence of MIH in the school population sampled was 21.5% and by severity 9.6% mild, 6.4% moderate, and 5.5% severe. Of the participants, 3.9% were underweight (weight-for-age) and 7.6% of low height (height-for-age), while, according to the BMI-for-age, 39.6% were normal, 23.6% were overweight, 28.7% were obese, and 8.2% were thin.  Table 1 presents the characteristics of the sample for children both with and without MIH. For all the school-age children, the presence of MIH was higher for the older age categories. In terms of gender, a higher percentage was found for boys than for girls (55.2% vs. 44.8%; *p* =0.490, respectively) not finding statistically significant differences. Of the children presenting thinness, 16.2% also presented MIH, and only 6.0% did not (*p*  < 0.001).

**Figure 1:**
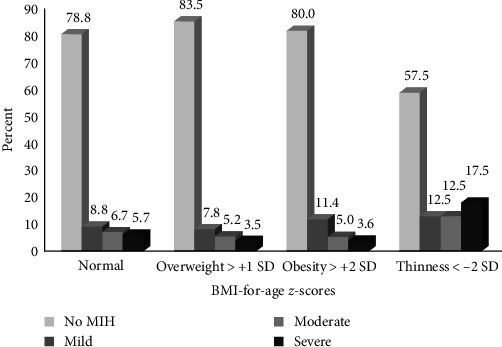
Percent distribution (%) between BMI-for-age *z*-scores and severity of molar–incisor hypomineralization (MIH) in schoolchildren 7–12 years old in Mexico (*n* = 488).

In general, 14 of the 362 (3.9%) school-age children aged 7–10 years were underweight (weight-for-age *z*-score < −2 SD), while 6.9% and 9.5% of the 7–10 and 11−12-year-old schoolchildren, respectively, were undersize (height-for-age *z*-score < −2 SD). Finally, 6.9% and 11.9% of the 7–10 and 11−12-year-old schoolchildren, respectively, presented thinness (BMI-for-age *z*-scores < −2 SD) (Table 2).  Figure 1 shows that the school-age children presenting thinness corresponded to a higher percentage of MIH in the severe category than those children of a normal weight (17.5% vs. 5.7%, *p*  < 0.001, respectively).

The logistic regression model, adjusted for gender, oral hygiene, F water concentration, number of erupted teeth, and BMI-for-age *z*-scores (Table 3), revealed that schoolchildren with thinness were more likely to present MIH [OR = 2.76 (CI95% 1.33–5.73); *p* = 0.006] than children with a normal BMI.

## 5. Discussion

The main findings of the present study showed an association between thinness and the presence of MIH in 7–12-year-old school-age children, using the WHO standards for growth, specifically the BMI-for-age *z*-scores. The results obtained suggest that MIH is related to malnutrition in school-age children. The BMI has become an anthropometric indicator widely recognized in clinical and epidemiological practice and enables the classification of children into categories of thinness, overweight, and obesity [27]. Various studies have found an association between being overweight and obesity and both DDE [28] and dental caries [29, 30]. Malnutrition and its subtypes have been associated with the presence of oral diseases. For example, DDE [31], caries [32], and dental fluorosis [33] have been associated with both stunted growth (low height) and low weight. With regard to thinness and its relationship with oral diseases, Shen et al. [34], in children aged 24–71 months, found that those presenting severe caries were more likely to present thinness (OR: 4.08). Although thinness (or underweight) could affect the child's general health, it has been subject to less research [35]. For example, thinness may present when children lack sufficient energy and protein, while protein-energy malnutrition (PEM) is associated with other nutritional problems, such as deficiencies in the micronutrients indispensable to healthy development [36]. Therefore, diagnosing thinness at an early age would assist in controlling the child's growth and nutritional status, thus avoiding problems with cognitive development and academic performance, and helping to reduce the occurrence of disease.

Malnutrition produces changes during the mineralization or maturation stage of amelogenesis and, as a result, produces defective and poorly calcified enamel. Studies have observed that malnourished populations present a higher percentage of enamel hypoplasia caused by a perturbation in the maturation of ameloblasts [37]. Malnutrition can both lead to a complete interruption of the secretory phase of the ameloblasts and produce a qualitative defect of the enamel [37]. Moreover, vitamin A deficiency has been associated with alterations in the formation and development of teeth [38], while there is also a possible link between both pre- and postnatal vitamin D deficiency and the presence of MIH [39]. Vitamin D stimulates the mineralization of enamel and bone, bonding with the receptors that are expressed in both dental and bone cells [40]. It has also been shown that PEM affects the patterns of dental eruption and produces a hypomineralized enamel [41].

Nutritional status may exert a strong influence on the formation of teeth, while malnutrition at a young age may affect tooth formation and result in a fragile and hypomineralized enamel with a higher susceptibility to caries [42]. Therefore, adequate nutrition during childhood growth and development substantially contributes to maintaining both general and oral health.

On the other hand, in present study, a prevalence of thinness of 8.2% was found; data in Mexico have reported prevalence of thinness between 3% and 7% in children between 6 and 11 years [43]. In various countries, nutritional inequalities vary according to ethnic origin, degree of urbanization, health inequities, and changes in socioeconomic conditions [44]. Likewise, malnutrition can affect the most vulnerable population and present negative consequences such as a decrease in the child's ability to resist infections and diseases; it also causes lifelong physical and cognitive damage. Finally, malnutrition could have a negative impact on economic and social capital [45]. Therefore, preventing malnutrition among vulnerable groups must be a priority. It is important to design health programs that address nutritional status from malnutrition to being overweight at the individual, family, and social level with the purpose of improving children's integral health.

The present study did not find a relationship between fluoride concentration in the drinking water and the presence of MIH. Similarly, the prevalence of MIH found in the present study was 21.5%, which is similar to that reported by other studies conducted on different fluoride concentrations in drinking water [46, 47]. The differences found in the prevalence of MIH by studies conducted on different fluoride concentrations may be attributed to the corresponding age groups; the sample size; the diagnostic criteria; the diversity of environmental, demographic, and climatic conditions; and the use of different indexes for evaluating MIH.

On the other hand, etiology of MIH is not yet defined, but congenital, systemic, and environmental factors that affect amelogenesis in early maturation stage or in late secretory phase have been proposed [48]. Although in the present study no significant association was found between fluoride and the presence of MIH, longitudinal studies are required to evaluate the effect that fluoride has on formation of teeth with MIH.

In the present study, oral hygiene was not found to be related to the presence of MIH. It has been reported that teeth with severe MIH are more susceptible to dental caries, due to their having certain characteristics, such as an irregular surface, greater porosity, and a tooth loss structure causing exposure of dentin once the tooth has been in contact with masticatory forces. Consequently, areas are formed that allow a greater accumulation of biofilm, making it difficult to remove during tooth brushing, causing a greater number of caries lesions on affected teeth [49].

The child population, especially in early stages, is more likely to present some type of malnutrition with a negative impact on their physical, mental, and social well-being; in Mexico, according to the results of ENSANUT, chronic malnutrition had a significant decrease from 26.9% to 13.6% [50]. Despite the reduction in the prevalence of malnutrition, this is not enough. It is necessary to develop public health strategies that supervise actions and programs to reduce child malnutrition. It would also be important to investigate the causes and factors associated with the reduction of chronic malnutrition in Mexico with the purpose of reducing the consequences that arise with short- and long-term health.

One of the limitations of the present study was its cross-sectional design, which does not enable the determination of the cause–effect relationship between the independent variables and MIH. Secondly, data corresponding to the prenatal and postnatal risk factors related to the presence of MIH were not collected. One advantage of the present study is that it is the first systematic review to examine the relationship between thinness, as measured using the WHO growth standards, and the presence of MIH. Therefore, the results obtained highlight the need for longitudinal studies that show how the risk factors interact with nutritional status in children with MIH.

## 6. Conclusions

An 8.2% prevalence of thinness was found by the present study, which also found it to be associated with the presence of MIH in school-age children aged 7–12 years. Malnutrition has negative consequences on not only tooth development but also health in general. Strategies and interventions are required to prevent and control micronutrient deficiencies in the child population. These strategies and interventions must include an adequate level of monitoring of the excessive consumption of micronutrients and should also mainly target vulnerable groups. Similarly, the timely diagnosis of MIH will contribute to the formulation of preventive noninvasive treatments that help to improve the oral health of the population.

## Figures and Tables

**Table 1:** Demographic and clinical characteristics in schoolchildren with and without molar–incisor hypomineralization (MIH) (*n* = 488).

Variables	No MIH *n* = 383 *n* (%)	MIH *n* = 105 *n* (%)	*p* ^*∗*^
Age			
7–8 years	83 (21.7)	24 (22.8)	0.156
9–10 years	208 (54.3)	47 (44.8)	—
11–12 years	92 (24.0)	34 (32.4)	—
Sex			
Boys	197 (51.4)	58 (55.2)	0.490
Girls	186 (48.6)	47 (44.8)	—
Oral hygiene (OHI-S)			
Good hygiene	174 (45.4)	38 (36.2)	0.091
Poor hygiene	209 (54.6)	67 (63.8)	—
Number of erupted teeth			
<23 teeth	115 (30.0)	27 (25.7)	0.389
≥23 teeth	268 (70.0)	78 (74.3)	—
Water F concentration			
1.00 ppm	233 (60.8)	68 (64.8)	0.463
1.40 ppm	150 (39.2)	37 (35.2)	—
Weight-for-age *z*-score ^*∗∗*^			
Normal	280 (96.2)	68 (95.8)	0.861
Underweight < −2 SD	11 (3.8)	3 (4.2)	—
Height-for-age *z*-score			
Normal	355 (92.7)	96 (91.4)	0.665
Low height < −2 SD	28 (7.3)	9 (8.6)	—
BMI-for-age *z*-scores			
Normal	152 (39.7)	41 (39.0)	<0.001
Overweight > +1 SD	96 (25.1)	19 (18.1)	—
Obesity > +2 SD	112 (29.2)	28 (26.7)	—
Thinness < −2 SD	23 (6.0)	17 (16.2)	—

^*∗*^Chi-square test; BMI, body mass index-for-age; SD, standard deviation.  ^*∗∗*^Weight-for-age indicator are not available for children in this age group and cannot be generated using the WHO *z*-scores (*n* = 362).

**Table 2:** Classification the nutritional status recommended by the WHO using *z*-score in schoolchildren 7–12 years-old in Mexico.

Variable	7–10 years *n* = 362 *n* (%)	11–12 years *n* = 126 *n* (%)
Weight-for-age *z*-score		
Normal	148 (40.9)	N/A
Overweight > +1 SD	95 (26.2)	N/A
Obesity > +2 SD	105 (29.0)	N/A
Underweight < −2 SD	14 (3.9)	N/A
Height-for-age *z*-score		
Normal	337 (93.1)	114 (90.5)
Low height < −2 SD	25 (6.9)	12 (9.5)
BMI-for-age *z*-scores		
Normal	144 (39.8)	49 (38.9)
Overweight > +1 SD	87 (24.0)	28 (22.2)
Obesity > +2 SD	106 (29.3)	34 (27.0)
Thinness < −2 SD	25 (6.9)	15 (11.9)

N/A, weight-for-age indicator are not available for children in this age group and cannot be generated using the WHO *z*-scores; SD, standard deviation.

**Table 3:** Adjusted odds ratios from the logistic regression model for molar–incisor hypomineralization (MIH) and predictors variables in schoolchildren 7–12 years of age in Mexico (*n* = 488).

Variables	Odds ratio crude (95% CI)	*p*	Adjust odds ratio (95% CI)	*p*
Sex^‡^	0.85 (0.55 – 1.32)	0.490	0.87 (0.55 – 1.36)	0.545
Oral hygiene (OHI-S)^¶^	1.46 (0.93 – 2.29)	0.092	1.41 (0.89 – 2.23)	0.140
Water F concentration^§^	0.84 (0.53 – 1.32)	0.464	0.71 (0.44 – 1.15)	0.167
Number of erupted teeth ^*∗*^	1.01 (0.98 – 1.05)	0.330	1.01 (0.97 – 1.04)	0.475
BMI-for-age *z*-scores^∞^				
Overweight > +1 SD	0.73 (0.40 – 1.33)	0.313	0.72 (0.39 – 1.32)	0.298
Obesity > +2 SD	0.92 (0.54 – 1.58)	0.782	0.88 (0.50 – 1.52)	0.654
Thinness < −2 SD	2.74 (1.33 – 5.06)	0.006	2.76 (1.33 – 5.73)	0.006

OR, odds ratio; CI, confidence interval. Reference group: sex^‡^, boys; oral hygiene^¶^, good; water F concentration^§^, 1.00 ppm; number of erupted teeth ^*∗*^, continuous variable; BMI-for-age *z*-scores^∞^, normal log likelihood = −246.21786; Hosmer–Lemeshow = 0.740.

## Data Availability

The database used to support the findings of this study is available from the corresponding author upon request.
